# Socio-demographic disparities in global trends of lip and oral cavity neoplasms from 1990 to 2021

**DOI:** 10.1038/s41598-025-88684-z

**Published:** 2025-02-04

**Authors:** Amr Sayed Ghanem, Ágnes Tóth, Attila Csaba Nagy

**Affiliations:** 1 Department of Health Informatics, Faculty of Health Sciences, University of Debrecen, Debrecen, Hungary; 2 Department of Integrative Health Sciences, Faculty of Health Sciences, University of Debrecen, Debrecen, Hungary

**Keywords:** Disease burden, Global burden of disease, Inequalities, Sociodemographic, Oral cavity, Cancer, Neoplasm, Joinpoint regression

## Abstract

Oral cancer, the 13th most common globally, is primarily squamous cell carcinoma linked to tobacco, alcohol, and HPV. Despite advances in care, it remains a major health concern due to high mortality and its impact on quality of life. This study analyzed socio-demographic disparities in oral cancer burden using data from 1990 to 2021. We analyzed annual incidence, mortality, and DALYs across 204 countries, using age-standardized rates and the Socio-demographic Index (SDI) to assess development-related impacts. Statistical methods included Kruskal–Wallis tests, linear regression, joinpoint regression for trends, and Exponential Smoothing for forecasts (2022–2030), with analyses conducted in STATA and Python, and *p* < 0.05 as significant. Incidence was highest in high SDI countries, while mortality and DALYs were highest in low and middle SDI countries (*p* < 0.001). From 1990 to 2021, incidence increased (AAPC: 0.5–1.0%), while mortality (APC: − 0.5%) and DALYs (APC: − 0.6%) declined in low SDI regions. Significant disparities across SDI categories (*p* < 0.001) showed incidence rising with socio-demographic development (R^2^ = 0.102, *p* < 0.001), with high-middle SDI regions bearing the highest mortality and DALYs. These findings highlight the need for awareness, prevention, early detection, and accessible care, especially in lower SDI regions.

## Introduction

Oral cancer including the lip and oral cavity cancers is a type of head and neck (HNC) cancer^1^, a malignant neoplastic disease^2^ and it is the 13th most common cancer globally^3^. Oral cavity cancer subsites are the anterior tongue, floor of mouth, buccal mucosa, hard palate, alveolar ridges retromolar trigone and gingiva. Common signs of lip and oral cavity cancers are sores or lumps on the lips or inside the mouth^4^. The major histologies of oral cavity cancers (approximately 90%) are squamous cell carcinomas, while other types are minor malignancies of the salivary glands, sarcomas, malignant odontogenic tumours, melanoma and lymphoma^5^.

According to the World Health Organization (WHO), the incidence of cancers of the lip and oral cavity over the world was approximately 377 713 new cases, which resulted in 177 757 deaths in 2020^3^ drawing attention to a mortality rate of close to 50%. Oral cancer is more frequent and deadly among men and older people compared to women^3^ and to younger population^3,6^. Early diagnosis is crucial for better prognosis and for reducing lethal cases^7^. Visual and palpation inspection are the most conventional diagnostic methods followed by biopsy and histopathological examination in case of patients with identified lesion. Imaging and a number of other techniques (e.g. toluidine staining) are used to contribute to precise detection and for staging the primary tumor(s) or regional lymph nodes^8^. Despite of the easy detection, many patients seek medical care only in advanced stages. The standard treatment is primary surgical resection with or without postoperative adjuvant therapy^9^ e.g. radiation and/or chemotherapy^10^.

Tobacco, areca nut/betel quid and alcohol use are considered to be the leading etiological factors of oral cavity cancers. In addition, the common sexually transmitted human papillomavirus (HPV) infections are accountable for the growing numbers of oral cancers among young people^3^. As smoking and other forms of tobacco use had become a global epidemic by the 1990s, the World Health Assembly approved the World Health Organization Framework Convention on Tobacco Control (WHO FCTC) on 21 May 2003 that entered into force on 27 February 2005^11^. Since its entry, the FCTC has made significant improvements in tobacco control but there have been wide variations between countries and policy areas. In general, there has been great advances in tobacco product labelling, training, education, public awareness and marketing restrictions to minors. The FCTC has also made significant progress on tobacco prices and taxes, advertising, promotion, cessation and surveillance^12,13^. In 2006, the US Food and Drug Administration (FDA) has approved the first HPV vaccine (against types 6, 11, 16 and 18)—originally as a cervical cancer elimination tool—representing another milestone among preventive interventions. By 2019, one hundred countries have adopted the vaccine as a part of their national schedules. However, global disparities can be seen in uptake rates: low income and low-middle income countries show lower rates of HPV vaccination than high-income countries^14^. Socio-demographic disparities significantly affect the incidence and outcomes of lip and oral cavity cancers. Studies have shown that individuals in lower socio-economic groups are more likely to engage in high-risk behaviors such as tobacco and alcohol use, major contributors to these cancers^15^. Limited access to healthcare services in these populations often delays diagnosis, leading to poorer outcomes^16^. Global data from the Global Burden of Disease study show that countries with lower socio-demographic indices (SDI) experience higher mortality rates from lip and oral cavity cancers, despite lower incidence rates compared to higher SDI countries^17^. This pattern highlights that while the occurrence of these cancers may be lower in less developed regions, outcomes are more severe due to inadequate early detection and treatment services^18^. Research also reveals an increasing burden of these cancers in lower to middle SDI countries, driven by a combination of risk behaviors and limited healthcare infrastructure^17^.

Despite global efforts, oral cavity cancer is still a serious health concern, not only because of its severe mortality rate but also due to its substantial impact on quality of life along its psychological and physical consequences e.g. anxiety, lack of activity, mood disturbances and postoperative complications^19^. At country level, the economic burden as a consequence of both health care costs and productivity losses of lip and oral cavity cancers is also substantial, especially, when the malignancy is detected in later stages^20^. Given at the disease shows significant variations along socio-economic circumstances^3^, the goal of this study was to assess socio-demographic disparities in the global burden of lip and oral cavity neoplasms and their impacts on incidence, mortality and disability-adjusted life years (DALYs) the time-based measure combining years of life lost due to premature mortality (YLLs) and the years lived with a disability (YLDs)^21^ based on the data of Global Burden Disease (GBD) over the period between 1990 and 2021. To the authors’ knowledge, this is the first study to assess the burden of lip and oral cavity cancers using the 2021 Global Burden of Disease (GBD) dataset, providing the most up-to-date insights into global trends. Highlighting socio-demographic disparities is crucial for identifying high-risk populations and addressing inequities in incidence, mortality, and DALYs. By filling a critical evidence gap, this study aims to inform targeted interventions and equitable healthcare strategies to mitigate these disparities.

## Materials and methods

### Study design

The annual incidence, mortality, and DALYs rates for lip and oral cavity neoplasms were estimated using data from the GBD 2021 study. Data were obtained from 1990 to 2021 from the publicly available Global Health Data Exchange database (https://vizhub.healthdata.org/gbd-results/, accessed on 19 February 2024)^22^. The GBD 2021 study provides comprehensive insights into the health burden of 369 diseases and injuries across 204 countries and territories, categorized by age groups, sexes, countries, and regions^23^. This dataset is available on global and national levels annually, covering the period from 1990 to 2021.

Data from 204 countries were collected using the GBD 2021 database to assess the burden of lip and oral cavity neoplasms. This burden was quantified in terms of incidence, mortality, and DALYs. Age-standardized incidence rates (ASIR), age-standardized mortality rates (ASMR), and age-standardized DALYs rates were collected globally and nationally from 1990 to 2021. These rates are expressed per 100,000 population, allowing for consistent comparisons over time and across different population sizes. Age-standardized rates were used in the analyses to account for changes in population size and age structure over the study period. The use of DALYs as a measure enabled capturing the comprehensive health impact of lip and oral cavity neoplasms by YLL due to premature mortality and YLD. This metric is essential for understanding both the quantity and quality of life affected by these neoplasms. The GBD 2021 study offers a detailed methodology for calculating these rates, which is documented extensively in GBD publications^24,25^. This methodology ensures robust and comparable estimates, which are critical for informing health policies, guiding international health organizations, and aiding in health system planning. By leveraging this comprehensive dataset, the current study provides information about the trends and impacts of lip and oral cavity neoplasms over the past three decades.

### Socio-demographic Index (SDI)

Developed by GBD researchers, the SDI is a composite indicator designed to assess development status and its correlation with health outcomes. The SDI is calculated as the geometric mean of three indices: total fertility rate under the age of 25 (TFU25), mean education for those aged 15 and older (EDU15 +), and lag distributed income (LDI) per capita. This composite measure ranges from 0 to 1, where an SDI of 0 represents the lowest possible level of development relevant to health, and an SDI of 1 represents the highest possible level. The SDI is strongly correlated with health outcome variables such as mortality, life expectancy, and DALYs, making it a valuable tool for predicting and comparing regional health outcomes. Countries are categorized into five SDI levels based on their scores (low SDI, low-middle SDI, middle SDI, high-middle SDI and high SDI). The five SDI categories include countries such as Ethiopia and Yemen in the low SDI group, Egypt and India in the low-middle SDI group, Brazil and Vietnam in the middle SDI group, Hungary and Turkey in the high-middle SDI group, and the United States and Japan in the high SDI group. These categories allow for a clear comparison of health outcomes across different levels of development, providing information on how socio-economic factors influence the burden of diseases. The data for SDI can be accessed from the Global Health Data Exchange database^26^.

### Statistical methods

A Kruskal–Wallis test was conducted to evaluate differences in age-standardized incidence, mortality, and DALYs across SDI categories, followed by Dunn’s test for post-hoc pairwise comparisons. The unit of analysis was each country or territory. To explore the relationship between age-standardized measures of lip and oral cavity neoplasms and the level of socio-demographic development, linear regression analysis and scatter plots were employed.

Joinpoint regression analysis was performed to detect significant changes in trends for the ASIR, ASMR, and age-standardized DALYs rate over the period from 1990 to 2021. The maximum number of joinpoints was set to three, and significant changes in the rates over time were assessed using the permutation test. *p*-values for the permutation tests were estimated using Monte Carlo methods^27^. The annual percent change (APC) for each segment and the average annual percent change (AAPC) as a weighted average of the APCs for the entire period were calculated. APC is used to characterize trends in rates over time by indicating a constant percentage change from the previous year’s rate, while AAPC provides an average of the APCs over multiple years. All joinpoint analyses were performed using the Joinpoint Regression Program (Version 5.1.0.0—April 2024) from the National Cancer Institute, Bethesda, MD, USA^28^. Statistical analyses were conducted using STATA IC version 17.0 software^29^. A *p*-value of less than 0.05 was considered statistically significant.

The graphs, including scatter plots, time series plots, and box plots, were generated using Python with various libraries^30^. Scatter plots were created using the ‘*Matplotlib’* and ‘*Seaborn’* libraries, while time series plots and box plots were also generated using ‘*Matplotlib’*.

For the forecasting of age-standardized incidence, mortality, and DALYs rates from 2022 to 2030, the Exponential Smoothing (ETS) method was employed. This statistical technique is well-suited for time series data and is effective in accounting for trends and seasonal variations and was already used in previous studies employing the same database^31–33^. The ETS method involves applying weighted averages to past observations, where more recent observations are given higher weights. The Holt-Winters implementation of the ETS model was used, which includes components for level, trend, and seasonality. Each of the historical data series (incidence, mortality, and DALYs) was fitted separately for different SDI Index levels, including global, low SDI, low-middle SDI, middle SDI, high-middle SDI, and high SDI categories. The forecasts were generated for a nine-year period (2022–2030), and the models were validated through visual inspection of the fitted values and residual diagnostics to ensure accuracy and reliability. All analyses were conducted using the Python programming language with the ‘*statsmodels’* library.

## Results

### Trends in disease burden of lip and oral cavity neoplasms in years 1990 to 2021

Globally, the ASIR increased from 4.27 (95% UI: 4.10–4.45) per 100,000 to 4.88 (95% UI: 4.52–5.20) per 100,000. In the low SDI category, the incidence rate initially increased, peaking at 2.91 (95% UI: 2.16–3.79) per 100,000 in 2014, before fluctuating and ultimately decreasing to 2.39 (95% UI: 1.72–3.22) per 100,000 in 2021. The low-middle SDI category saw an increase from 3.01 (95% UI: 2.57–3.51) per 100,000 in 1990 to a peak of 3.41 (95% UI: 2.51–4.27) per 100,000 in 2021. The middle SDI category exhibited fluctuations, with an increase from 4.92 (95% UI: 4.27–5.69) per 100,000 in 1990 to a peak of 5.21 (95% UI: 4.56–5.94) per 100,000 in 1995, and then decreasing to 2.95 (95% UI: 2.26–4.84) per 100,000 in 2021. In the high-middle SDI category, the rate grew from 5.41 (95% UI: 4.94–5.92) per 100,000 in 1990 to a peak of 6.7 (95% UI: 4.37–5.26) per 100,000 in 2007 before decreasing to 4.84 (95% UI: 3.99–5.79) per 100,000 in 2021. Lastly, the high SDI category showed an increase from 4.12 (95% UI: 3.55–4.74) per 100,000 in 1990 to a peak of 5.30 (95% UI: 4.78–5.86) per 100,000 in 2013 before decreasing to 4.71 (95% UI: 4.05–5.43) per 100,000 in 2021. Conversely to the global ASIR, the global ASMR decreased from 2.45 (95% UI: 2.33–2.58) per 100,000 to 2.42 (95% UI: 2.23–2.60) per 100,000. In the low SDI category, the rate decreased from 2.02 (95% UI: 1.55–2.60) per 100,000 in 1990 to 1.86 (95% UI: 1.34–2.48) per 100,000 in 2021, despite fluctuations and a peak of 2.28 (95% UI: 1.70–2.96) per 100,000 in 2014. The low-middle SDI category experienced an increase from 2.06 (95% UI: 1.76–2.40) per 100,000 in 1990 to 2.40 (95% UI: 1.78–3.16) per 100,000 in 2021, with some fluctuations over the years.

**Fig. 1:**
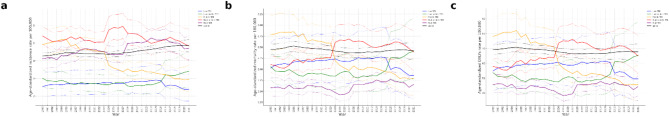
Time trends in (**a**) age-standardized incidence rates, (**b**) age-standardized mortality rates, and (**c**) age-standardized DALYs rates of lip and oral cavity neoplasms from 1990–2021. Dashed lines show the 95% uncertainty intervals.

The middle SDI category saw a decrease from 2.79 (95% UI: 2.41–3.23) per 100,000 in 1990 to 1.79 (95% UI: 1.40–2.26) per 100,000 in 2021, reaching a peak of 2.85 (95% UI: 2.49–3.24) per 100,000 in 1995. In the high-middle SDI category, despite some fluctuations over the years, showed a steady general increase as the rate rose from 2.10 (95% UI: 1.93–2.28) per 100,000 in 1990 to 2.39 (95% UI: 1.97–2.87) per 100,000 in 2021, with a peak of 2.68 (95% UI: 2.36–3.04) per 100,000 in 2007. Lastly, the high SDI category showed a slight increase from 1.58 (95% UI: 1.38–1.80) per 100,000 in 1990 to 1.65 (95% UI: 1.41–1.92) per 100,000 in 2021, despite fluctuations and a peak of 1.76 (95% UI: 1.58–1.93) per 100,000 in 2013. In terms of DALYs, the global rate showed a slight decrease from 69.27 (95% UI: 65.92–72.96) in 1990 to 67.71 (95% UI: 61.32–73.17) in 2021. The low SDI category saw the DALYs rate rise from 54.86 (95% UI: 41.28–71.88) in 1990 to 61.44 (95% UI: 44.90–81.13) in 2014 followed by a sharp decrease reaching 49.55 (95% UI: 34.90–67.68) in 2021. For low-middle SDI, it decreased from 53.74 (95% UI: 45.59–63.07) in 1990 to 47.27 (95% UI: 37.88–59.14) in 2009, then a rise followed, reaching its peak in 2021 65.40 (95% UI: 47.27–87.83). The middle SDI category saw a general decrease from 78.16 (95% UI: 67.27–91.21) to 45.59 (95% UI: 34.71.56–58.88) between 1990 and 2021. In high-middle SDI regions, DALYs rose from 57.86 (95% UI: 53.16–62.99) to 76.22 (95% UI: 66.99–86.81) in 2007 then decreasing to 64.96 (95% UI: 52.96–78.77) in 2021. Lastly, the high SDI category experienced an increase from 45.83 (95% UI: 40.19–52.30) in 1990 to 44.63 (95% UI: 51.69–37.89) in 2021 with some marked fluctuations in between (Fig. 1a–c).

### Burden of lip and oral cavity neoplasms according to SDI

**Fig. 2:**
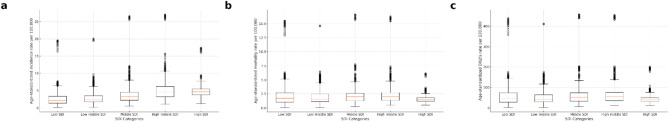
Distribution of age-standardized incidence (**a**), mortality (**b**) and DALYs (**c**) across the five SDI categories.

The distribution of age-standardized rates of oral and lip neoplasms across SDI groups is displayed in Fig. 2a–c The Kruskal–Wallis test demonstrated significant differences in age-standardized rates of incidence, mortality, and DALYs for lip and oral cavity neoplasms across SDI groups from 1990 to 2019, with all *p*-values < 0.001.

For incidence, Dunn’s pairwise comparisons revealed significant differences between Low SDI and Low-Middle SDI (mean rank difference =  − 4.238, *p* < 0.001), Low SDI and Middle SDI (-16.80, *p* < 0.001), Low SDI and High-Middle SDI (-31.40, *p* < 0.001), and Low SDI and High SDI (-27.40, *p* < 0.001). Other significant differences included Low-Middle SDI and Middle SDI (-12.70, *p* < 0.001), Low-Middle SDI and High-Middle SDI (-27.00, *p* < 0.001), Low-Middle SDI and High SDI (-24.00, *p* < 0.001), Middle SDI and High-Middle SDI (-13.30, *p* < 0.001), and Middle SDI and High SDI (-12.80, *p* < 0.001). The difference between High-Middle SDI and High SDI was not significant (-1.50, *p* = 0.067).

For mortality, Dunn’s pairwise comparisons showed significant differences between Low SDI and Low-Middle SDI (1.692, *p* = 0.045), Low SDI and Middle SDI (-4.833, *p* < 0.001), Low SDI and High-Middle SDI (-7.112, *p* < 0.001), and Low SDI and High SDI (5.871, *p* < 0.001). Additionally, significant differences were found between Low-Middle SDI and Middle SDI (-6.233, *p* < 0.001), Low-Middle SDI and High-Middle SDI (-8.452, *p* < 0.001), Low-Middle SDI and High SDI (4.580, *p* < 0.001), Middle SDI and High-Middle SDI (-2.119, *p* = 0.017), and Middle SDI and High SDI (9.119, *p* < 0.001). The difference between High-Middle SDI and High SDI was also significant (10.828, *p* < 0.001).

For DALYs, Dunn’s pairwise comparisons indicated significant differences between Low SDI and Low-Middle SDI (2.769, *p* = 0.009), Low SDI and Middle SDI (-4.473, *p* < 0.001), Low SDI and High-Middle SDI (-7.871, *p* < 0.001), and Low SDI and High SDI (5.551, *p* < 0.001). Significant differences were also observed between Low-Middle SDI and Middle SDI (-6.814, *p* < 0.001), Low-Middle SDI and High-Middle SDI (-10.10, *p* < 0.001), Low-Middle SDI and High SDI (3.477, *p* < 0.001), Middle SDI and High-Middle SDI (-3.109, *p* = 0.001), and Middle SDI and High SDI (8.545, *p* < 0.001). The difference between High-Middle SDI and High SDI was significant (11.109, *p* < 0.001) (Table 1).

**Table 1:** Kruskal–Wallis test and Dunn’s pairwise comparisons for age-standardized rates of incidence, mortality, and DALYs of lip and oral cavity neoplasms across Socio-Demographic Index groups (1990–2021).

Metric	Kruskal–Wallis test results	Pairwise comparisons	Mean rank difference	Adj. Sig
Incidence	** < 0.001**	Low SDI to Low-Middle SDI	− 4.238	** < 0.001**
		Low SDI to Middle SDI	− 16.8	** < 0.001**
		Low SDI to High-Middle SDI	− 31.4	** < 0.001**
		Low SDI to High SDI	− 27.4	** < 0.001**
		Low-Middle SDI to Middle SDI	− 12.7	** < 0.001**
		Low-Middle SDI to High-Middle SDI	− 27	** < 0.001**
		Low-Middle SDI to High SDI	− 24	** < 0.001**
		Middle SDI to High-Middle SDI	− 13.3	** < 0.001**
		Middle SDI to High SDI	− 12.8	** < 0.001**
		High-Middle SDI to High SDI	− 1.5	0.067
Mortality	** < 0.001**	Low SDI to Low-Middle SDI	1.692	**0.045**
		Low SDI to Middle SDI	− 4.833	** < 0.001**
		Low SDI to High-Middle SDI	− 7.112	** < 0.001**
		Low SDI to High SDI	5.871	** < 0.001**
		Low-Middle SDI to Middle SDI	− 6.233	** < 0.001**
		Low-Middle SDI to High-Middle SDI	− 8.452	** < 0.001**
		Low-Middle SDI to High SDI	4.58	** < 0.001**
		Middle SDI to High-Middle SDI	− 2.119	**0.017**
		Middle SDI to High SDI	9.119	** < 0.001**
		High-Middle SDI to High SDI	10.828	** < 0.001**
DALY	** < 0.001**	Low SDI to Low-Middle SDI	2.769	**0.003**
		Low SDI to Middle SDI	− 4.473	** < 0.001**
		Low SDI to High-Middle SDI	− 7.871	** < 0.001**
		Low SDI to High SDI	5.551	** < 0.001**
		Low-Middle SDI to Middle SDI	− 6.814	** < 0.001**
		Low-Middle SDI to High-Middle SDI	− 10.1	** < 0.001**
		Low-Middle SDI to High SDI	3.477	** < 0.001**
		Middle SDI to High-Middle SDI	− 3.109	**0.001**
		Middle SDI to High SDI	8.545	** < 0.001**
		High-Middle SDI to High SDI	11.109	** < 0.001**

Bold values indicated statistical significance *p* < 0.05, lower mean rank difference is indicated by a negative number. Abbreviations: Socio-demographic Index (SDI).

**Fig. 3:**
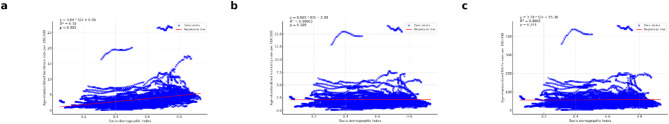
Association between Socio-demographic Index and age-standardized incidence (a), mortality (b) and DALYs (c) rates.

The linear regression analysis for the age-standardized incidence rates (ASIR) of lip and oral cavity neoplasms revealed a significant association with the SDI. The model showed an R squared of 0.102, indicating that approximately 10.17% of the variability in ASIR can be explained by SDI (*p* < 0.001). For mortality rates, the regression model did not find a significant association with SDI (R squared = 0, *p* = 0.569). Lastly, for DALYs, the model also did not show a significant relationship with SDI (R squared = 0.0002, *p* = 0.243). These results indicate a significant positive association between SDI and incidence rates but no significant association between SDI and mortality or DALYs for lip and oral cavity neoplasms (Fig. 3a-c).

### Joinpoint regression analysis of disease burden by SDI

For incidence, Low SDI saw an initial increase (APC = 0.391%) until 2014, followed by a significant decline (APC =  − 2.742%), resulting in an overall AAPC of − 0.325%. Low-middle SDI experienced a significant initial decrease (APC =  − 0.553%) until 2011, then a significant rise (APC = 2.996%), with an overall AAPC of 0.578%. Middle SDI consistently decreased (AAPC =  − 1.766%), while High-middle SDI had mixed trends, ending with a slight decrease (AAPC =  − 0.269%). High SDI showed a significant initial increase (APC = 1.482%) and another during 2005–2013 (APC = 2.612%), followed by a decrease (APC =  − 1.510%), with an overall AAPC of 0.514%.

For mortality, Low SDI began with an increase (APC = 0.297%) and then saw a significant reduction (APC =  − 3.081%), resulting in an AAPC of − 0.476%. Low-middle SDI had a significant initial decrease (APC =  − 0.326%) followed by an increase (APC = 3.379%), with an overall AAPC of 0.736%. Middle SDI experienced significant decreases during two periods (APC =  − 0.719% and − 6.753%) and a rise during 2006–2012 (APC = 0.437%), leading to an overall AAPC of − 1.570%. High-middle SDI showed mixed trends, ultimately increasing (AAPC = 0.597%). High SDI exhibited a slight initial increase (APC = 0.201%) and a significant rise during 2005–2013 (APC = 2.688%), but an overall AAPC of 0.125%.

For DALYs, Low SDI began with an increase (APC = 0.253%) and then experienced a significant decrease (APC =  − 3.278%), resulting in an AAPC of − 0.556%. Low-middle SDI had a significant initial decrease (APC =  − 0.305%) followed by increases (APC = 7.426% and 1.959%), with an overall AAPC of 0.782%. Middle SDI consistently declined (AAPC =  − 1.865%), while High-middle SDI had mixed trends, ending with an overall increase (AAPC = 0.577%). High SDI initially decreased (APC =  − 0.450%) but had mixed trends, ultimately resulting in a slight decrease (AAPC =  − 0.091%).

### Forecasted trends in age-standardized incidence, mortality, and DALYs rates (2022–2030)

**Fig. 4:**
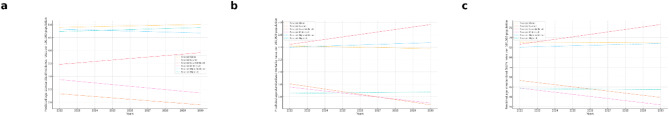
Forecasted age-standardized incidence (**a**), mortality (**b**) and DALYs (**c**) rates (2022–2030) by SDI levels.

The forecasted age-standardized rates for incidence, mortality, and DALYs from 2022 to 2030 demonstrate varying trends across different SDI levels. Incidence rates are projected to increase globally, with the highest increases observed in the low-middle SDI group, while high SDI regions show a more gradual rise. Mortality rates are predicted to decline slightly across all SDI levels. DALYs rates exhibit a mixed pattern, with some SDI levels showing a decrease, particularly in low and middle SDI groups, while high-middle SDI regions experience a modest increase. The detailed forecasted values are summarized in Table 3 and Fig. 4a–c.

## Discussion

Over the past 30 years or so, life expectancy at birth on the global scale has increased progressively: while the average for both sexes was 64 years in 1990, this demographic indicator has risen to 72 in 2020^34^. Further increase is already visible for the present and anticipated for the future^35^. The growing ratio of the elderly contributes to the emergence of an ageing society. However, longer life does not always imply healthy life; the incidence rate of many diseases increases as a function of age, as well as the incidence of cancer rate^36^. In fact, age can be considered as a major risk factor for cancer overall^37^ and for several individual cancers including lip and oral cavity carcinomas. The incidence rate accelerates after the age of 50, especially for adults aged 65 and over^38^. Since oral cancer is a multifactorial disease^39^, the objective of this study was to analyse the annual incidence, mortality and DALYs for lip and oral cavity neoplasms worldwide in an age-standardized manner to exclude the impact of age and to focus on the possible effects of socio-demographic disparities by means of SDI categories developed by Global Burden Disease researchers for 204 countries over the period between 1990 and 2021.

### Incidence of oral cancer

Our linear regression model, outlined in Fig. 3, indicated that about 10.17% of the observed variability in age-standardized incidence rates (ASIR) can be attributed to changes in SDIs, confirming the association between ASIR of lip and oral cavity neoplasms and SDI categories. Other risk factors may include lifestyle, environmental and occupational factors^40^ related to socio-demographic variables^41–43^. Furthermore genetic factors, family history of cancer^44,45^, immunosuppression^46^ etc. can also play a role. The most important lifestyle risk factors are consumption of tobacco, betel quid/areca nut chewing^47^ and alcohol use, which in combination with tobacco acts synergistically in the development of oral cavity cancers^48^. Other behaviour risk factors including unhealthy diet and nutrition, obesity^49^ can be also responsible for the development of oral cavity cancers. Poor oral hygiene and health^44,45^, chronic intraoral inflammation such as periodontitis^50^, chronic mechanical stress^51^ and recurrent oral ulcers^52^ were also shown to increase the susceptibility for oral cavity cancer. Among different types of infections, the HPV subtypes 16, 18, 31, 33 and 35^53^ are the main contributors in epithelial cell transformation and oral carcinogenesis^47,54^. In terms of environmental exposures, ultraviolet (UV) radiation can cause lip cancers^54^. In low-middle income countries, occupational exposures are more frequent^55^. Due to the habit of smokeless tobacco, areca nut chewing, oral cancer is the most common malignancy in South-, South-East Asia and in the Western Pacific islands such as India, Sri Lanka Bangladesh or Papua New Guinea^47,56^, which regions were ranging mainly in low and low-middle to middle SDIs within the period from 1990 to 2021 according to GBD^26^.

In general, the risk of developing oral cancer is lower in people with higher levels of education and income^45^. Yet, our analysis revealed a monotonically increasing distribution of ASIR of lip and oral cavity neoplasms in the five different SDI groups from low towards the high SDI countries. Our pairwise comparisons of SDI groups, as shown in Table 1, demonstrated significant differences in ASIR across each SDI groups with the exception of the difference of ASIR between high-middle and high SDI regions but a slightly upward trend is still detectable. It may be attributed to the phenomenon that although tobacco and alcohol use were decreasing in the past few decades, HPV prevalence was found to be increasing around the world^2^. According to a systematic review of population based studies by Menezes et al., the incidence of HPV-related head and neck cancer (HNC) subsites elevated, while the HPV-unrelated subsites diminished or remained stable. An increase in HPV-related cases was detected in Canada, United States of America, Hong Kong and Korea^57^ ranging between high-middle and high SDI categories over the past three decades based on GBD data^26^. The incidence trends were increased for both HPV-related and HPV-unrelated cases in Peru ranging from low-middle to middle SDIs, in Taiwan showing a continuous increase in SDI from middle to high and in European countries (e.g. Denmark, England, Norway, The Netherlands)^57^ belonging to the high-middle and mostly to the high SDI category^58^ over the period between 1990 to 2021^26^. The increasing tendency of ASIR towards higher SDI categories can be also explained by previous findings: individuals with lower socio-economic status are less likely to participate in cancer screening programs compared to people with higher socio-economic status^59^, in addition, residents in poor countries have less access to health services than those in wealthier territories^60^, thus, a higher number of oral cancer cases can be diagnosed in counties with higher SDIs compared to those with lower SDIs.

Our time-series analysis, presented in Fig. 1, detected an overall increase in ASIR globally from 1990 to 2021. Analyzing temporal trends in this time period, as detailed in Table 2, we detected a significant increase between 1990 and 2014 and a significant decrease between 2014 and 2021 in the low SDI category. We observed a significant decrease between 1990 and 2011 and a significant increase between 2011 and 2021 in the low-middle category. A significant decline was found in the period between 1990 and 2002 in the middle SDI group, followed by an even more dramatic decline between 2002 and 2005. After a significant increase from 2002 to 2005, a significant decline was detectable from 2005 to 2021 in the high-middle category. ASIR in the high SDI group exhibited significant increases in the periods from 1990 to 2000 and from 2005 to 2013, then a decline was shown from 2013 to 2021. Despite fluctuations, the overall trends over the total examined period of 1990 and 2021 were declining in the low, middle and high-middle SDI countries, whereas increasing trends were observed in the low-middle and high SDI countries. FCTC and/or the of HPV vaccination might have been played a role in several declines after 2005 and 2006, however, since cancer develops over years or decades and the impacts can be seen only on long term^61^, therefore the exact causative links resulting joinpoints are challenging to prove.

**Table 2:** Joinpoint regression analysis of temporal trends in mortality, incidence, and DALYs rates for lip and oral neoplasm across Socio-demographic Index categories (1990–2019).

	Trend 1		Trend 2		Trend 3		Trend 4		1990–2021
	Period	APC (%)	Period	APC (%)	Period	APC (%)	Period	APC (%)	AAPC (%)
Incidence									
Low SDI	1990–2014	0.391*	2014–2021	− 2.742*					− 0.325*
Low-middle SDI	1990–2011	− 0.553*	2011–2021	2.996*					0.578*
Middle SDI	1990–2002	− 0.920*	2002–2005	− 7.809*	2005–2021	− 1.226*			− 1.766*
High-middle SDI	1990–2002	− 0.355	2002–2005	4.610*	2005–2021	− 1.094*			− 0.269*
High SDI	1990–2000	1.482*	2000–2005	− 1.447	2005–2013	2.612*	2013–2021	− 1.510*	0.514*
Mortality									
SDI Low	1990–2014	0.297*	2014–2021	− 3.081*					− 0.476*
SDI Low-Middle	1990–2012	− 0.326*	2012–2021	3.379*					0.736*
SDI Middle	1990–2002	− 0.719*	2002–2006	− 6.753*	2006–2012	0.437	2012–2021	− 1.657*	− 1.570*
SDI High-Middle	1990–2002	0.756	2002–2005	5.757*	2005–2021	− 0.459*			0.597*
SDI High	1990–2001	0.201	2001–2005	− 2.847*	2005–2013	2.688*	2013–2021	− 0.995*	0.125
DALYs									
SDI Low	1990–2014	0.253*	2014–2021	− 3.278*					− 0.556*
SDI Low-Middle	1990–2013	− 0.305*	2013–2016	7.426	2016–2021	1.959			0.782*
SDI Middle	1990–2002	− 0.888*	2002–2005	− 8.412*	2005–2021	− 1.323*			− 1.865*
SDI High-Middle	1990–2002	1.046	2002–2005	5.775	2005–2021	− 0.715			0.577*
SDI High	1990–2001	− 0.45	2001–2006	− 2.691*	2006–2009	7.445*	2009–2021	− 0.479*	− 0.091

Statistically significant results are indicated by an asterisk (*), significance is *p* < 0.05, Abbreviations: Socio-demographic Index (SDI), annual percent change (APC) average annual percent change (AAPC).

Based on our current projections, as shown in Table 3 and Fig. 4, the ASIR trends for the low and medium SDI groups are expected to continue to decline between 2022 and 2030, while that of the low-middle SDI group is expected to continue to increase. The ASIR of high-middle and high SDI groups are predicted to be statistically stable, similarly to the global trend. In the future, trends might be influenced by further health politics including vaccination strategies, the changes in public awareness, in tobacco and alcohol using habits and the presence of other individually modifiable risk factors of oral cancer.

**Table 3:** Forecasted values of age-standardized incidence, mortality and DALYs rates from 2022 to 2030.

Year	Global	Low SDI	Low-middle SDI	Middle SDI	High-middle SDI	High SDI
Incidence						
2022	4.894	2.328	3.463	2.875	4.823	4.733
2023	4.908	2.275	3.52	2.811	4.805	4.753
2024	4.922	2.222	3.576	2.747	4.787	4.772
2025	4.935	2.168	3.633	2.682	4.768	4.791
2026	4.949	2.115	3.69	2.618	4.75	4.81
2027	4.963	2.062	3.746	2.553	4.732	4.83
2028	4.977	2.008	3.803	2.489	4.713	4.849
2029	4.99	1.955	3.86	2.425	4.695	4.868
2030	5.004	1.902	3.916	2.36	4.677	4.888
Mortality						
2022	2.419	1.808	2.445	1.755	2.4	1.655
2023	2.414	1.766	2.485	1.723	2.409	1.658
2024	2.409	1.723	2.525	1.691	2.418	1.66
2025	2.404	1.681	2.565	1.658	2.427	1.662
2026	2.399	1.639	2.605	1.626	2.437	1.665
2027	2.393	1.596	2.645	1.594	2.446	1.667
2028	2.388	1.554	2.686	1.561	2.455	1.67
2029	2.383	1.511	2.726	1.529	2.464	1.672
2030	2.378	1.469	2.766	1.497	2.473	1.674
DALYs						
2022	67.672	48.295	66.651	44.45	65.194	44.129
2023	67.631	47.224	67.899	43.392	65.423	44.075
2024	67.589	46.153	69.147	42.333	65.653	44.021
2025	67.547	45.083	70.395	41.275	65.882	43.967
2026	67.506	44.012	71.644	40.217	66.111	43.912
2027	67.464	42.941	72.892	39.159	66.341	43.858
2028	67.422	41.87	74.14	38.1	66.57	43.804
2029	67.381	40.799	75.388	37.042	66.799	43.75
2030	67.339	39.728	76.637	35.984	67.028	43.696

### Mortality of oral cancer

Oral cancer is a life-threatening disease. Early detection has an utmost significance in better survival of oral cancer^7^ as late-stage disease contributes to high mortality^62^.

Our pairwise comparisons of SDI groups, as depicted in Table 1, revealed significant differences in age-standardized mortality rates (ASMR), however, in contrast to the distribution of ASIR, ASMR distribution was heterogeneous, therefore no statistically significant trend was observed along SDIs. Yet, the lowest ASMR was detected in the highest SDI countries despite of their highest incidence rate, which might be reasonable by the observations that individuals of higher socio-economic status display higher likelihood in attending cancer screenings^59^ and due to enhanced financial and human resources and services, these populations are in a much more advantageous positions in terms of access to health care^60^ including prevention, diagnosis and treatment^63^. For instance, salivary biomarkers enable disease detection even in the case of asymptomatic individuals providing opportunities for earlier detection and for reduction of mortality^62^. In addition, new types of treatments, such as immunotherapy including monoclonal antibodies^64^ and immune checkpoint inhibitors^65^ are being developed from clinical trials, which can dramatically improve the treatment outcomes, however immunotherapy is extremely expensive^66^ which also contributes to inequalities in accessibility.

Our time-series analysis, as displayed in Fig. 1, showed a slight global decrease in ASMR in the examined period between 1990 and 2021, which can be attributable to the advances in medical interventions in head and neck cancers including oral cavity neoplasms^67^. Temporal increase and decrease in ASMR was detected in the low SDI group from 1990 to 2014 and from 2014 to 2021, respectively, while in the low-middle SDI group a temporal decrease from 1990 to 2012 was followed by a significant increase from 2012 to 2021, as shown in Table 2. The middle SDI group performed several declines over the three decades including periods from 1990 to 2002, from 2002 to 2006 and from 2012 to 2021, of which the decline between 2002 and 2006 was the most substantial. A significant rise was detected from 2002 to 2005 in the high-middle SDI group, which was followed by a slight but still significant decrease from 2005 to 2021. From 2001 to 2005, a decrease in ASMR was observed in the high SDI category, which was followed by rise from 2005 to 2013 and a decrease from 2013 to 2021. Over the total examined period of 1990 to 2021, with all the fluctuations in ASMR, along with the observed decreasing incidence rates, the low and middle SDI countries showed decreasing trends as well in mortality rates, whereas the low-middle SDI countries displayed increasing mortality rate with the observed increase in incidence rate. The high SDI category remained statistically stable over the 30-year period despite fluctuations in ASMR and the increasing overall ASIR, which might be linked to early detection possibilities and the available effective interventions. Interestingly, in spite of the overall decrease in ASIR, ASMR was found to be elevated in the high-middle SDI category between 1990 and 2021. Furthermore, the mortality rate was the highest in this SDI group. Although detailed epidemiological analyses are needed to determine the exact causalities, it may refer to late-stage diagnosis, when the outcome is less favourable or to regional disparities in these populations.

After the overall rise in ASMR between 1990 and 2021 in the high-middle SDI group, our forecast analysis predicts stability for the period between 2022 and 2030, similar to the ASMR of the high SDI countries, as displayed in Table 3 and Fig. 4. The global ASMR tendency is expected to be statistically stable as well. Further declines in ASMR are predicted in the low and middle SDI groups, whereas increases in ASMR is predicted in the low-middle SDI category. Early diagnosis and the elimination of disparities in access would be essential to improve survivorships in the future^66^.

### DALYs of oral cancer

In addition to premature death, the disease can cause serious consequences in quality of life as well. Its primary treatment is surgery, when the aim is to resect the tumor with a margin of the healthy tissue. Sometimes reconstructive operation is need to minimize the morbidity of the resection^5^. However, even with a successful reconstruction, there may remain dysfunctions in speech, chewing, swallowing, there can be changes in the patient’s appearance and the risk of airway obstruction may cause short- or long-term tracheostomy and feeding tube dependence^10,19^. The quality of perioperative care is crucial to ensure positive outcomes for the oncological patients^5^.

Assessing the impact of oral cancer on DALYs, similarly to mortality rates, we revealed mixed distribution along different SDI groups. Our pairwise comparisons showed significant differences between each SDI group, outlined in Table 1. Besides the lowest mortality rate, the lowest DALYs was found in the high SDI category, which might be in line with better accessibility to health care facilities and with the possibilities of earlier detection in countries with high SDIs. Besides the reduction of mortality rate, early-stage detection has critical role in reduction of possible complications as well, leading to better outcome and prognosis; delays in diagnosis may involve more intensive treatments, more loss of function and morbidity^7,68^. In addition, the end-of-life care is better in countries with high SDIs^63^. The high-middle SDI group had showed the highest mortality rates and it appeared with the highest DALYs data as well, which is presumably related to later stages of cancer detection^62^.

In the period from 1990 to 2021, our time-series analysis, shown in Fig. 1, displayed similarities between DALYs and overall mortality rates as well: slight decrease was detectable globally, the low and middle SDI countries also declined, the low-middle SDI countries appeared with increasing tendencies and the high SDI category remained stable despite temporal fluctuations, which appeared as follows: a significant rise was observed in DALYs in the low SDI group between 1990 and 2014, displayed in Table 2, which was followed by a decrease from 2014 to 2021. From 1990 to 2013, a decreasing trend was observed in the low-middle SDI group, then a dramatic increase was detected from 2013 to 2016. Countries in the middle SDI category performed several significant decays over the examined three decades, from 1990 to 2002, from 2002 to 2005, being the most prominent one, and from 2005 to 2021. DALYs in the high-middle SDI category increased substantially from 2002 to 2005, while DALYs in the high SDI group appeared with significant decrease from 2001 to 2006, a dramatic increase from 2006 to 2009 and another significant decrease from 2009 to 2021. DALYs for lip and oral cavity cancers in low and low-middle SDI regions reflect limited healthcare access, late diagnoses, and high-risk behaviors like smokeless tobacco and betel quid chewing^69,70^. Declines in low SDI DALYs post-2014 align with tobacco control measures under WHO FCTC which also resulted in a steady decrease of DALY in the middle SDI category^71^. In low-middle SDI regions, economic growth has increased tobacco and alcohol use, while inadequate healthcare and low HPV vaccination rates have driven DALYs upward post-2013^72^. In high-middle and high SDI regions, the rise in DALYs between 2002 and 2009 aligns with increasing HPV-associated oral cancers, a growing contributor in these populations^73^. The subsequent decline in DALYs from 2009 onwards likely reflects the impact of preventive measures, including widespread HPV vaccination programs and strengthened public health efforts to reduce tobacco and alcohol use^74^. These interventions have contributed to improved detection, treatment outcomes, and a reduction in the overall disease burden in higher SDI regions.

Based on our projections for the period between 2022 and 2030, DALYs in the low and medium SDI groups are expected to continue to decline, further rise is predictable in the low-middle SDI category, whereas stability is expected in the high-middle and high SDI countries similarly to the global trend. Further positive changes should be achieved in the future by enhancing public awareness and by national screening programs targeting especially high risk populations.

### Strengths and limitations

A key strength of this study is its analysis of global patterns and trends in the burden of lip and oral cavity neoplasms across a long time span and different development categories. The population-based data provides an in-depth look into global disparities. However, the joinpoint regression results may vary depending on parameter settings and data volume. National-level analysis may mask within-country differences, and variations in data collection, lifestyle, and healthcare access could impact data consistency. Additionally, the aggregated nature of the data limits the inclusion of individual clinical factors like tumor stage and treatment outcomes. Despite these limitations, the study effectively highlights important global trends and inequalities in the burden of lip and oral cavity neoplasms.

## Conclusion

Analyzing data provided by GBD, our study assessed an increasing trend in the age-standardized incidence rate globally with slightly decreasing trends in the age-standardized mortality rate and in the disability-adjusted life years linked to lip and oral cavity cancer. At the same time, significant variability was observed in the burden of the disease along countries with different SDIs. Improved public awareness, elimination of modifiable risk factors would be crucial for prevention, as regular oral check-ups for early-stage detection would facilitate the better outcomes and improved survivorship of the disease.

## Data Availability

Publicly available datasets were analyzed in this study. Data can be accessed and downloaded using the following link: https://vizhub.healthdata.org/gbd-results/ (accessed on 19th of May 2024).
